# The motivations and experiences of specialists who provide outreach services in rural operating rooms: A survey study from British Columbia

**DOI:** 10.1371/journal.pone.0298757

**Published:** 2024-03-27

**Authors:** Anshu Parajulee, Kathrin Stoll, Nancy Humber, Sean Ebert, Kim Williams, Jude Kornelsen

**Affiliations:** 1 Department of Family Practice, University of British Columbia, Vancouver, British Columbia, Canada; 2 Rural Coordination Centre of British Columbia, Vancouver, British Columbia, Canada; Government Institute of Medical Sciences, INDIA

## Abstract

**Introduction:**

Outreach care has long been used in Canada to address the lack of access to specialist care in rural settings, but research on the experiences of specialists providing these services is lacking. This descriptive survey study aimed to understand 1) specialists’ motivation for engaging in outreach work, (2) their perceptions of the quality of care at their rural outreach hospital, and (3) the supports they receive for their outreach work, in order to create a supportive framework to encourage specialist outreach contributions.

**Methods:**

In July 2022, specialist physicians who provide outreach operating room services at rural hospitals participating in the Rural Surgical and Obstetrical Networks initiative in the province of British Columbia were invited to complete an anonymous survey.

**Results:**

21 of 45 invited outreach specialists completed the survey (47% response rate). Three-quarters of respondents had a surgical specialty. The opportunity to deliver care to underserved patients was the most common motivator for outreach work. Rural hospitals received high ratings from respondents on overall safety and various aspects of communication and teamwork. Postoperative care was a concern for a minority (one-fifth) of respondents, and about half had experienced unnecessary delays between procedures some or most of the time. Generally, respondents felt integrated into rural teams and reported receiving adequate nursing and anesthetic support. The two most common desired additional supports were better/more equipment and space and additional staffing. All 19 respondents not planning to retire soon intended to provide outreach services for at least three more years.

**Conclusion:**

Specialists providing outreach OR services in small volume rural hospitals in BC usually have altruistic motives for outreach work. For the most part, these specialists have positive experiences in rural hospitals, but they can be better supported through investment in infrastructure and health human resources. Specialists intend to provide outreach services long-term, indicating a stable outreach workforce. More research on the facilitators and barriers of specialist outreach work is needed.

## Introduction

Rural communities across Canada, and in other jurisdictions internationally, contend with challenges in access to specialist care due to low population density, which does not support focused practice [1]. Globally, a central contributing factor to the lack of equitable healthcare access for rural residents is deficiency in the numbers and types of healthcare providers in rural areas [2]. This demands that patients travel from their home communities, sometimes long distances, to access such care, creating logistical and financial challenges [3, 4] and conflicting with the policy mandate of care “closer to home” [5, 6]. This can lead to lower levels of access despite higher levels of need for some services in rural communities [7]. In a tiered health care system, resources are distributed based on anticipated population-level needs, starting with generalist care in low volume communities and triage pathways to higher levels of specialist care when required [8]. Interventions to mitigate the need for patient travel to access specialist care include virtual care when appropriate and outreach services from specialists in regional referral centres. The World Health Organization recommends outreach services as a strategy to address the shortage of healthcare providers, including specialists, in rural areas [2].

Models of specialist outreach in the province of British Columbia (BC) include supported travel from a regional centre to rural sites for patient consults and low acuity procedures. The efficacy of this model depends on both the capacity of the specialist to travel to provide care and the resources available at the rural site to support the care, for example, anesthesia, nursing, and recovery support. However, little is known about the antecedents and motivations of specialists who provide outreach care or the qualities of rural sites most conducive to supporting this care. This paper examines both of these considerations in an effort to identify evidence-based interventions to better support outreach specialist services in rural settings.

### Access to specialist care in rural Canada

Canadian provider distribution data show that 18% of the population live rurally [9] but only two percent of specialist physicians are based in a rural jurisdiction [10]. Population need dictates limited local specialist practice in communities <20,000 due to a lack of procedural volume to support a full practice [11]. For a community to be a main practice location, a specialist needs to be able to perform a minimum number of procedures in that community to maintain their skillset and obtain a sufficient personal income. This limitation is reflected in data from BC, which shows four percent of general surgeons, three percent of obstetricians, and three percent of orthopedic surgeons are rurally based [12]. The low prevalence of rural specialists gives rise to the need for outreach services, defined in this context as care by providers who have a main practice in one community and provide regular in-person services in another (rural) community. Rates of outreach work in Canada are unknown; in comparison, one in every five specialist physicians provide outreach services in Australia, a jurisdiction with similar healthcare delivery models [13, 14] and comparable rurality [15, 16].

### Benefits of outreach specialist services

There is limited Canadian data on outreach specialist care. A study from rural Australia demonstrated that compared to out-of-town access, local access to specialist care via outreach clinics is associated with increased completion of specialist referrals and a decrease in hospital admissions [17]. In-person follow-up gives specialists the opportunity to improve patient outcomes by addressing post-operative issues; the rate of follow-up appointment completion may be lower if out-of-town patient travel is required [4, 18]. Rural patients in Canada can also experience considerable cost savings as a result of outreach services, as demonstrated by costing modeling for outreach orthopedic surgery clinics in remote Labrador [19] and outreach rehabilitation medicine clinics in remote Manitoba [20]. Total transportation and accommodation cost savings, which took into account both costs saved for patients and costs incurred for specialists, was C$2,185 per patient visit in 2019/20 for the Manitoba clinics and C$1,080 per patient visit in 2019 for the Labrador clinics. This substantially mitigates patient out-of-pocket costs to access care which, in BC, was shown to be C$2,044 on average per health condition [3].

Outreach specialists who perform procedural care in rural operating rooms (ORs) not only reduce the need for rural residents to travel but also play an integral role in sustaining and strengthening rural OR’s by contributing to the threshold volumes required for sustainability [21]. Concomitantly, this can have a positive impact on the rural OR team through confidence-building due to increased procedural volume, mentorship during participation in a wider slate of procedures, and opportunities for improved team function. Taken together, these benefits lead to increased satisfaction among local healthcare providers [17, 22, 23]. An additional positive consequence of cross-regional relationships is the opportunity to improve relationships between regional and referral sites across all care-provider domains (proceduralists, nurses), and site-level efficiencies in ensuring regional patient needs are met [17, 23].

### Motivations for outreach practice

Motivations for engaging in outreach work illustrate the potential ‘wins’ for specialists. Some motivators, such as increasing patient volume relate to individual career goals while other motivators, such as the opportunity to practice in underserved communities, reflect a responsibility to contribute to sustainable regional services [24, 25]. Outreach work can also help specialists better understand rural contexts, enabling them to provide better care and improve patient flow (e.g., discharge planning) for rural patients [4, 23].

There are also, however, disincentives to engaging in outreach work including travel times to rural sites and the attendant possibility of travel challenges due to inclement weather and poor road conditions. Long travel times and work hours associated with outreach work can disrupt work-life balance, increasing the risk of burnout [26, 27].

### Supports for outreach specialists

Australia, which has a national policy on subsidies for specialist rural outreach, has been promoting and supporting rural outreach services through its Rural Outreach Fund, implemented in 2000 [28]. The national government finances this program and collaborates with states and territories to distribute funds to specialists who receive reimbursement/remuneration for travel-related expenses, travel times, and other outreach related costs, such as cultural training, equipment rental, and administrative support [28, 29]. In Canada, governmental support for outreach specialists varies by province/territory. In BC, the Northern and Isolation Travel Assistance Outreach Program provides funding to healthcare providers for travel times and travel related expenses, including accommodation [30].

However, additional infrastructure supports are required, including well-resourced and highly functioning primary care services [4, 31]. Rural staff and physicians need to integrate outreach specialists into the local team [31] and hospitals need to provide the infrastructure, including equipment and staff, that specialists need to deliver high-quality care [32]. A high degree of interprofessional coordination and collaboration between rural hospitals, specialist offices, and Health Authorities is needed during the planning of outreach care. This can include triaging patients, arranging for specialist physician and nurse travel, and planning for collaborative surgeries by local family physicians with enhanced surgical skills and outreach specialists.

### Rural Surgical and Obstetrical Networks initiative

Canada’s publicly funded healthcare system provides universal coverage at no cost to patients for services deemed to be medically necessary [33]. Both the federal and provincial/territorial governments fund this not-for-profit system and share roles and responsibilities. Each province/territory has a separate public healthcare insurance plan and is responsible for organizing and delivering services for its residents. The province of BC is divided into five geographic Health Authorities, with each Health Authority responsible for planning and delivering healthcare services within its jurisdiction [34].

This study is part of the comprehensive evaluation of the Rural Surgical and Obstetrical Networks (RSON) initiative that was implemented in the province of BC. This initiative provided supports to rural hospitals in five domains: clinical coaching, continuous quality improvement, remote presence technology, scope and volume of procedures, and evaluation [35]. RSON was created in response to the destabilization of maternity and surgical programs in rural BC over the past three decades [35]. The eight rural hospitals that participated in RSON and received OR services from outreach specialists at the time of data collection will be referred to as ‘RSON hospitals’. Care in the OR at these hospitals is provided by a combination of outreach specialists and local healthcare providers comprised of family physicians with enhanced surgical skills, solo specialist physicians, and/or dentists.

Networks of clinical care across expansive rural geographies are essential to meet the comprehensive healthcare needs of rural residents. This requires a commitment to ensuring the right provider is providing the care in the right location. Supported outreach specialist models have been shown to be an effective way to do this both in BC and in other jurisdictions. For these models to thrive, we need to understand the experience of outreach providers. This study aimed to provide data to better support outreach care by understanding outreach specialists’ (a) motivations for engaging in outreach work, (b) perceptions of the quality of care at their outreach hospital, and (c) supports they receive for their outreach work.

## Methods

### Study design

This was a cross-sectional exploratory survey study conducted in rural communities in BC. Because rural outreach specialist services have not been investigated in depth in BC before, we chose to use a survey method to collect data from a larger number of respondents about numerous aspects of outreach care. Qualitative interviews, which require more time from study participants, would have likely yielded a smaller response rate due to the limited availability of physicians. Furthermore, an anonymous survey can encourage more candid and valid responses, particularly for sensitive topics such as the perceived quality of care in rural hospitals.

### Survey tool

Survey items on the quality of care at RSON hospitals cover the areas of efficiency, safety, teamwork, communication, and pace/workload. Almost all the quality items are from one of three questionnaires developed by the Agency for Healthcare Research and Quality [36–38] in the United States. We reviewed these questionnaires and selected quality related items that visiting specialists, who are at outreach hospitals infrequently and for limited periods of time, would likely have knowledge of. Items on supports for outreach specialists were developed by the study team after reviewing peer-reviewed publications from Canada [26, 39], the United States [24, 40], the United Kingdom [41], and Australia [25, 31, 42, 43] about physicians who provide outreach or locum services. These publications also informed the development of items on respondents’ demographic characteristics and motivations for outreach work. Findings from interviews with rural healthcare providers and administrators, conducted as part of the larger RSON evaluation, also informed the development of survey items. We reviewed the interview data and identified any key aspects of outreach specialist services not already included in the draft survey tool, e.g., the proportion of OR equipment provided by outreach hospitals. The larger RSON evaluation examined various aspects of rural surgical and obstetrical networks including patient and provider experiences, patient health outcomes, and network costs. RSON program members, including RSON hospital representatives, reviewed the survey tool and provided their feedback.

### Survey administration and analysis

The anonymous survey was administered at the eight RSON hospitals that receive outreach OR services from specialist physicians. At each hospital, an RSON Local Community Coordinator or an OR staff member emailed a survey invitation in July 2022 and two reminders in August 2022 to eligible outreach specialists serving their hospital. To participate in the study, specialists needed to have provided outreach OR services for at least one year at an RSON hospital. Across RSON hospitals, the number of outreach specialists invited to the survey ranged from one to nine.

Staff at two hospitals, who thought that visiting specialists would likely complete a paper survey during their break time, recommended also administering a paper version of the survey. At these hospitals, specialists who chose to complete a paper survey placed a sealed envelope with their survey in a locked survey box or submitted their sealed envelope to the local staff member coordinating survey administration. Local staff collected sealed envelopes with completed paper surveys and mailed unopened envelopes to the study team. One hospital posted a paper survey advertisement that included a QR code. The survey was open for two months in 2022, from mid-July to mid-September. The online version was hosted on the University of British Columbia approved Qualtrics survey platform.

We also used data collected as part of the RSON initiative to understand RSON hospital characteristics. Descriptive statistical analyses were conducted in IBM SPSS Statistics Version 28 and Microsoft Excel. Results for survey items with >10% ‘does not apply or don’t know’ responses are not reported. All survey items were voluntary, and any missing values were excluded from analyses.

This survey study was funded by BC’s Joint Standing Committee on Rural Issues and received ethical approval from the University of British Columbia’s Behavioural Research Ethics Board (H21-03756). The survey consent form stated that electronic or paper submission of the survey signified that respondents had read the consent form and provided their consent to participate in the study. The study team did not have any information that could identify individual survey respondents during or after data collection. Only study team members based at the University of British Columbia in Vancouver, BC had access to data files. These team members are not healthcare providers or administrators and were not familiar with the identity or characteristics of visiting specialists at any of the rural RSON hospitals. As the survey was anonymous, respondents were not able to withdraw their participation after submitting their survey.

## Results

### RSON hospital characteristics

RSON hospitals have an annual OR volume of less than 700 and receive outreach services from between one and nine specialists (Table 1). The most common types of outreach OR procedures at RSON hospitals are endoscopes and dental, orthopedic, gynecological, plastic, ear nose and throat, and urologic surgeries. RSON hospitals serve approximately 5,000–14,000 local individuals who reside within a one-hour driving distance (i.e., catchment population) (Table 1). Note that ‘n’ in tables refers to the number of communities or respondents for which data were available.

**Table 1:** RSON hospital characteristics.

	Median	Range
Local one-hour drive time catchment population, 2016^a^ (n = 7)	8,219	5,285–13,778
Annual number of OR procedures, 2019^b^ (n = 7)	297	95–662
Number of outreach specialists who provided services in the OR at the time of survey administration (n = 8)^c^	5.5	1–9

^a^ Refers to the number of individuals who live within a one-hour drive time of RSON hospitals. Data obtained from Statistics Canada [44]. Data not available for one RSON hospital; the catchment size for this hospital should be within the range for the other seven RSON hospitals.

^b^ Data obtained from Population Data BC as part of the larger RSON evaluation. Data not available for one RSON hospital; the annual OR volume for this hospital should be within the range for the other seven RSON hospitals.

^c^ Data obtained from local staff at RSON hospitals.

### Respondent characteristics

Twenty-one of the 45 outreach specialists (47%) invited to complete the survey did so. Sixteen completed an online version of the survey while five completed a paper version. One of the 16 who completed an online version used a QR code to access the survey; the QR code was included in a paper flyer posted in one of the RSON hospitals administering a paper survey.

Three in four respondents had a surgical medical specialty (Table 2). The majority (76%) had completed medical school in Canada and the most common preferred gender pronoun was he/him (70%) (Table 2). Gender is a socially constructed phenomenon that can influence how individuals view themselves and others, and how they behave and interact with others [45]. As this is a behavioural study (not a clinical one), gender is a more relevant concept than biological sex. In Canada, preferred pronoun(s) correspond with one’s gender identity. Respondents who did not identify as a binary woman (‘she/her’) or man (‘he/him’) were able to select ‘they/them’ and/or ‘other’ gender pronouns.

**Table 2:** Respondent characteristics–Specialty, training location, and gender pronoun.

	n	%
Medical specialty (n = 21)		
Surgery^a^	16	76
Internal medicine^b^	2	10
Dental	2	10
Family medicine—Enhanced surgical skills	1	5
Location of medical school (n = 21)		
Canada	16	76
Outside of Canada	5	24
Gender pronoun(s) (n = 20)		
He/him	14	70
She/her	5	25
They/them	0	0
Other	1	5

^a^ Four respondents were orthopaedic surgeons and two were plastic surgeons.

^b^ One respondent was a gastroenterologist.

Respondents were at various stages of their career as physicians, with practice experience ranging from less than five years to more than 20 years (Table 3). Sixty percent of respondents had at least five years of rural outreach experience in Canada (Table 3). Forty percent had been providing outreach rural services in Canada for the entire duration of their practice career. About half of respondents had more than five years of experience as an outreach specialist at a rural RSON hospital.

**Table 3:** Respondent characteristics—Practice experience.

	n (%)			
	<5 years	5–10 years	11–20 years	20+ years
Physician in Canada (n = 21)	5 (24)	5 (24)	7 (33)	4 (19)
Outreach physician in rural Canada (n = 20)	8 (40)	4 (20)	5 (25)	3 (15)
Outreach physician at a rural RSON hospital (n = 21)	10 (48)	4 (19)	4 (19)	3 (14)

For about half of respondents, the one-way travel time from one’s place of residence to an RSON hospital was more than two hours. The median number of outreach visits to an RSON hospital over the past six months was six. Among respondents who had to travel out of town to an RSON hospital and had at least one visit in the past six months, the median number of days spent in an RSON community per visit was 2.5. Half of respondents saw at least 20 patients on average per outreach visit. Refer to Table 4 for more information on respondents’ outreach work.

**Table 4:** Respondent characteristics–Outreach work.

	Median	Range
One-way travel time to RSON hospital (hours) (n = 20)	2	0–6.0^a^
Number of outreach visits in the past six months (n = 19)	6	0–18^b^
Average number of days spent in RSON community per visit^b^^c^ (n = 18)	2.5	0–12
Average number of patients seen per visit^c^ (n = 20)	19	3–76

^a^ Two respondents live in the community where they provide outreach services (their main practice is in another community).

^b^ One respondent had not had an outreach visit to an RSON hospital in the past six months. This respondent did not answer any question that asked about outreach experience in the past six months.

^c^ Over the past six months.

### Motivations for outreach work

**Fig 1:**
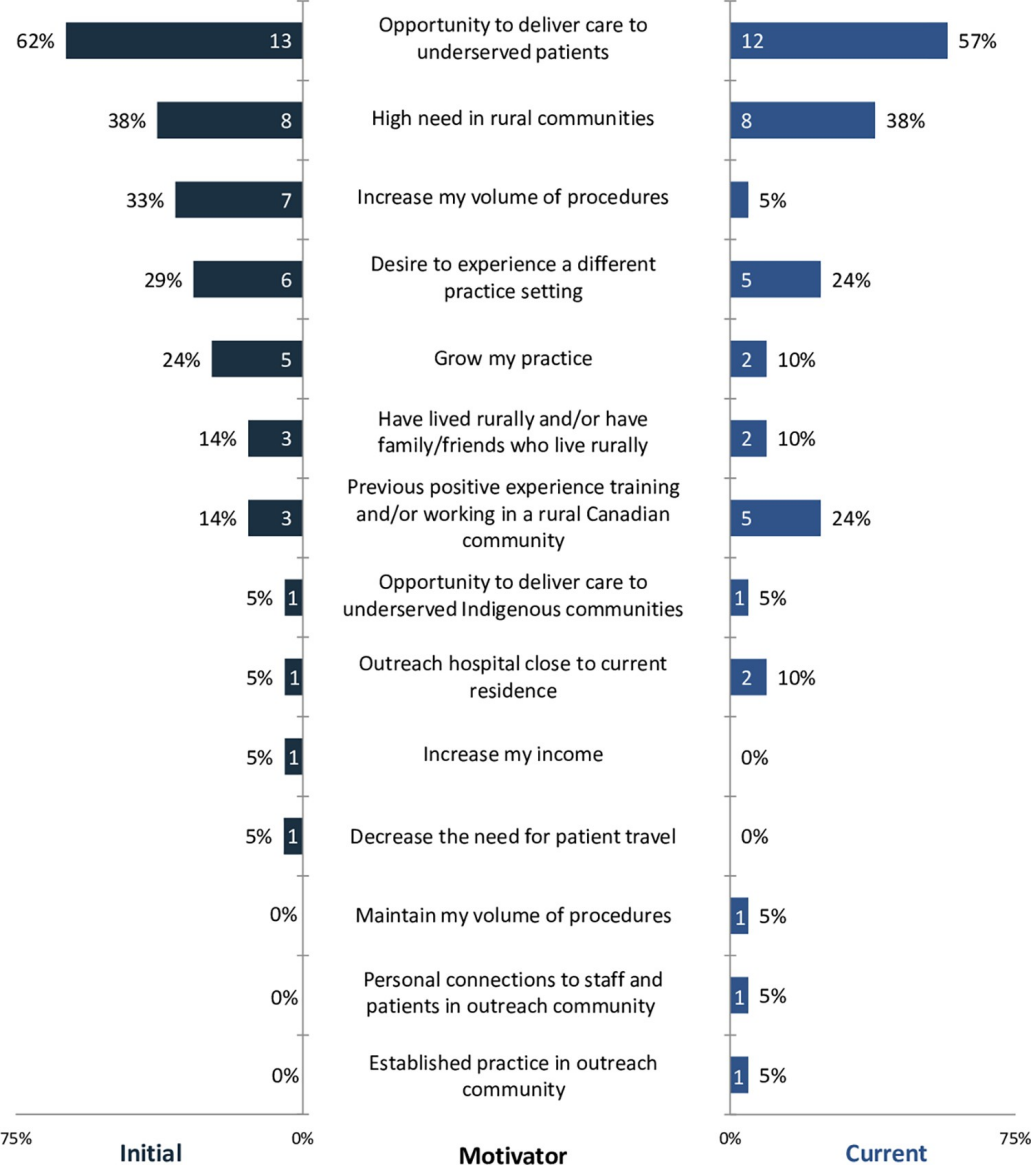
Most important motivators for providing outreach services initially and currently (21 respondents at each time point; numbers inside bars are counts).

Respondents were asked about their top motivators for providing outreach services at two time points: initially when they began providing outreach services and currently at the time of survey completion. The opportunity to deliver care to underserved patients was the most common motivator at both time points; it was a motivator for 62% of respondents initially and 57% of respondents currently (Fig 1). Some motivators were selected more often initially than currently, most notably increasing one’s volume of procedures and growing one’s practice, the benefits of which can include more procedural practice and more income. The respondents who selected either of these two as current motivators had six years of practice experience or less at the time of survey completion.

### Quality of care at RSON hospitals

Most respondents reported that in the past six months, RSON hospital team members had always or most of the time discussed (a) the overall plan of what was to be done before the start of procedures and (b) any concerns for patient recovery immediately after procedures (Table 5). A minority (38%) always had enough time between procedures to properly prepare for the next one (Table 5). Although all reported experiencing unnecessary delays, this occurred rarely for about half of respondents (Table 5). Most (86%) thought that their workload during their last outreach visit was ‘about right’ (Table 6). Although all respondents rated overall patient safety at their outreach RSON hospital as ‘good’, ‘very good’, or ‘excellent’, one-fifth had over the past six months been concerned about their outreach RSON hospital’s ability to provide post-operative care some or most of the time (Table 7).

**Table 5:** Quality of care at RSON hospitals–Communication and pace.

	Just before the start of the procedures, all team members stopped to discuss the overall plan of what was to be done. (n = 20)		Immediately after procedures, team members discussed any concerns for patient recovery. (n = 20)	
	n	%	n	%
Always	14	70	12	60
Most of the time	5	25	6	30
Sometimes	0	-	1	5
Rarely	0	-	0	-
Never	0	-	0	-
Does not apply or Don’t know^a^	1	5	1	5
	How often is there enough time between procedures to properly prepare for the next one? (n = 21)		How often do you experience unnecessary delays? (n = 21)	
	n	%	n	%
Always	8	38	0	-
Most of the time	9	43	1	5
Sometimes	4	19	9	43
Rarely	0	-	11	52
Never	0	-	0	-

^a^ One respondent who mostly provided services in a clinic in the six months before survey completion and had participated in a small number of procedures in the OR answered ‘does not apply or don’t know’ to both communication questions.

**Table 6:** Quality of care at RSON hospitals–Workload.

	How would you rate your workload during your last outreach visit to the RSON hospital? (n = 21)	
	n	%
Too much	0	-
About right	18	86
Too little	3	14

**Table 7:** Quality of care at RSON hospitals–Safety.

	I am concerned about the RSON hospital’s ability to provide post-operative care. (over the past six months) (n = 20)			Based on your experience, how would you rate the RSON hospital on overall patient safety? (n = 21)	
	n	%		n	%
Always	0	-	Excellent	9	43
Most of the time	1	5	Very Good	9	43
Sometimes	3	15	Good	3	14
Rarely	7	35	Fair	0	-
Never	9	45	Poor	0	-

**Fig 2:**
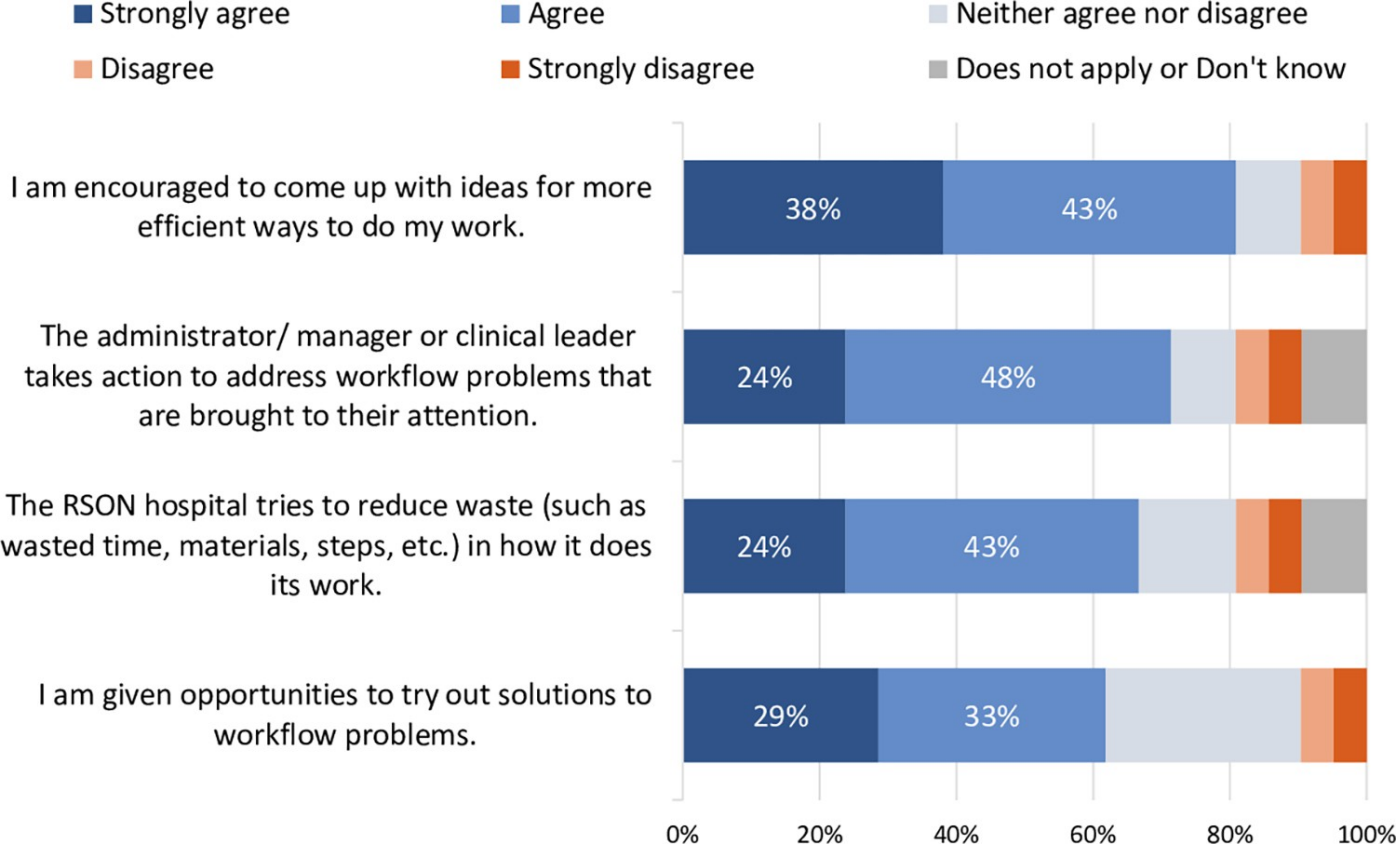
Quality of care at RSON hospitals–Efficiency (n = 21).

Almost all respondents (95%) agreed that team members at RSON hospitals anticipate each other’s needs and relay information in a timely manner (S1 Fig). At least 75% agreed with each of five other statements about teamwork: understanding one’s roles and responsibilities, delivering feedback in a constructive manner, ensuring availability of necessary resources, following a standardized method for sharing information when handing off patients, and holding each other accountable (S1 Fig). Most (81%) agreed that they are encouraged to come up with ideas for more efficient ways to do their work and a majority (>60%) agreed with each of three other statements about workflow or waste (Fig 2).

### Supports for outreach work

Respondents were asked about supports they had received as an outreach specialist at an RSON hospital over the past six months. Ninety percent agreed that they feel integrated into the RSON hospital team (S2 Fig). All agreed that team members are friendly and 90% agreed that team members are helpful (S2 Fig). Most (≥85%) agreed with each of three other statements about rural team integration: feeling appreciated, feeling comfortable sharing observations or concerns, and feeling that one’s ideas and suggestions are valued by the team (S2 Fig).

Almost all respondents received adequate nursing and anesthetic support most or all of the time; fewer (70%) received adequate administrative support most or all of the time (Table 8). Only about a third (30%) always had access to the medical equipment they needed to provide care (Table 9). For a majority of respondents (70%), all the medical equipment they use during procedures is provided by their outreach RSON hospital (Table 9). Most (80%) agreed that they had received adequate information/training on local policies and procedures (Table 10).

**Table 8:** Support during outreach work—Human resources.

I have adequate…	Nursing support (n = 20)		Anesthetic support (n = 20)		Administrative support (n = 20)	
	n	%	n	%	n	%
Always	13	65	16	80	11	55
Most of the time	6	30	3	15	3	15
Sometimes	0	-	0	-	3	15
Rarely	1	5	0	-	2	10
Never	0	-	0	-	0	-
Does not apply or Don’t know	0	-	1	5	1	5

**Table 9:** Support during outreach work–Equipment.

	I have access to the medical equipment that I need to provide care. (n = 20)		% provided by RSON hospital	On average, what percentage of the medical equipment that you use during surgeries/procedures is provided by the RSON hospital? (n = 20)	
	n	%		n	%
Always	6	30	100	14	70
Most of the time	13	65	90–99	3	15
Sometimes	0	-	75–89	2	10
Rarely	1	5	<75	1	5
Never	0	-			

**Table 10:** Support during outreach work–Training, compensation, and reimbursement.

	I have received adequate information/training on local policies and procedures. (n = 20)		I am satisfied with the compensation provided for my outreach services. (n = 20)		I am satisfied with the coverage/ reimbursement for indirect costs associated with my outreach work (n = 18)	
	n	%	n	%	n	%
Strongly agree	5	25	6	30	5	28
Agree	11	55	7	35	5	28
Neither agree nor disagree	2	10	3	15	3	17
Disagree	2	10	3	15	3	17
Strongly disagree	0	-	0	-	2	11
Does not apply or Don’t know	0	-	1	5	0	-

A minority (15%) were not satisfied with compensation for their outreach services (Table 10). Among respondents who traveled out of town to their outreach RSON hospital, 28% were not satisfied with coverage of/reimbursement for indirect costs associated with outreach work (Table 10).

Thirteen respondents listed various ways in which they could be better supported to provide high-quality patient care at their outreach RSON hospital. The most identified support (n = 6) was improved equipment and space—bigger and more “functional” perioperative space, a “better” space to see outpatients, and more or different surgical equipment. Two respondents were keen to provide a wider range of services at an RSON hospital but needed the equipment to do this.

The second most identified desired support was more staff (n = 4), specifically increased availability of clerical staff, surgical assists, or nurses. For one respondent, increased nursing support during anesthetic procedures in an outpatient clinic would allow for improved efficiency. The third most identified support was reimbursement for costs associated with outreach work (n = 3). Respondents described various challenges with reimbursement provided by the Northern and Isolation Travel Assistance Outreach Program, such as rules around which types of hotels are covered and difficulty tracking claims.

Two respondents were planning to retire soon; all the remaining 19 respondents were very likely to continue providing services at their outreach RSON hospital for the next three years.

## Discussion

Specialist outreach plays an essential role in promoting health equity for rural populations by improving access and mitigating delayed or abandoned care-seeking [4, 17, 18]. We can reasonably assume this leads to system cost-saving by mitigating the need for later, most costly intervention associated with disease progression. Beyond the social benefit of reducing the need for patient travel and reducing associated financial and social costs, outreach services stabilize rural sites by increasing local scope and volume, which in turn provides more clinical exposure for local teams (family physicians with enhanced surgical skills, family physician anesthetists, registered nurses). It also allows for integrated Continuing Professional Development and coaching through local participation in procedures that local providers would otherwise not be exposed to. Given the clear advantages of outreach care, it is crucial to build a supportive infrastructure to integrate regional care, including outreach care, into health services planning. Findings from this survey, reflecting specialists’ experiences in rural sites, are a step to understanding how this might be achieved.

Two fundamental qualities of rural sites that support outreach care are a *positive site culture* and *availability of resources*. For the first quality, most respondents in this study rated RSON hospitals highly on overall safety and various aspects of communication and teamwork, and also reported feeling well-integrated into the local teams. Additionally, most felt appreciated and found team members to be friendly and helpful. This contrasts with team experience more generally; a recent study in BC (2022) found only 63% of physicians reported feeling that they belong to a collaborative, patient-centered team/unit [46]. Contributors to the sense of positive team function in this study included activities such as the involvement of specialists in local continuous quality improvement processes and practicing in an environment where team members deliver feedback in a way that delivers positive interactions and future change. The latter aspect was consistent with reports of clear communication in the pre- and post-procedure period.

Perhaps the most salient indication of positive team culture, however, was a sense of team cohesion, based on reported qualities such as overall clarity of roles and responsibilities, the receptivity of site members to constructive feedback, and adherence to standardized procedures. Underscoring this more pragmatic functionality, however, were more general reports of affective dimensions of team cohesion: *friendliness* and *helpfulness*. These contributors to positive site culture were contingent in part on known relationships due to the low number of care providers at rural sites and the capacity to adapt to the needs and preferences of visiting specialists in ways that may be more difficult in larger sites. This encapsulates a *rural advantage*, that is, the contention that the dominant, largely deficit-dominant portrayal of health services delivery in rural communities is not only *not* based in evidence on safety and quality of care [47], but in fact misses the myriad of advantages of rural health service delivery. That is, due to smaller low populations and consequent potential for relationships between care providers and patients to exist outside the clinical encounter, more personalized patient-centred care has been noted [48]. Likewise, as active members of the community themselves, health care providers may have a better understanding of community needs, resulting in more targeted health care programming [49]. From a health service delivery perspective, due to personal associations between care providers and administers and comparatively reduced bureaucracy, service delivery models care pivot with more agility in response to changing circumstances (such as the COVID-19 pandemic) [50, 51]. Ultimately, perhaps, is the tendency of rural communities to develop innovative solutions to health service challenges *out of necessity*: that is, when there is the lack of a local safety net and the next health service is hours away, there is more motivation to develop local solutions [52].

As noted above, an additional essential quality of rural sites supporting outreach specialist care is the availability of the resources necessary to enable quality care. Most respondents reported having access to adequate resources. Respondents in this study identified specific areas where resources were adequate (such as nursing and anaesthesia support) but also areas where limited resources constrained their capacity to provide the level of care they aspired to. This included local availability of equipment and space, lack of the former limiting the scope of procedures that could be provided. More tangible implications of gaps in site resources, however, were concerns from some respondents about adequate post-operative care, reflecting the lack of staff and other resources for step-down units and inpatient post-operative care. Adequate resourcing reflects decisions made at a regional level, ideally guided by the goal of meeting population requirements for health care in the most efficient way possible. Achieving this goal demands conceptualizing specialist departments at regional centres as having the responsibility to support care across the region in a distributed, community-responsive way.

Even when positive site culture is developed and resources are bolstered to meet expanded care needs, there are pragmatic challenges to supporting outreach care. These primarily include difficult logistics of travel due to unpredictable weather over challenging geographies and the attendant lifestyle and resource implications of this travel. Although there are subsidies available to support the costs of travel and missed opportunities for clinical income through the Northern and Isolation Travel Assistance Outreach Program in the study jurisdiction [30], approximately one third of respondents in this study (28%) felt the program was inadequate in covering costs incurred. Among this minority, there were also frustrations with the administrative process of submitted claims. Addressing these issues are crucial for long-term program contribution as approximately half of the respondents in our study had a one-way travel time to an ROSN hospital of at least two hours.

Stabilizing and sustaining procedural care across a distributed geography is contingent on two commitments: (1) supporting and sustaining local resources and processes at the rural site and (2) creating a practice pathway for the participation of regional specialists that provides benefit for both rural and regional sites. The latter requires attention to optimizing rural outreach work setting by ensuring adequate case load volume, clearly articulated roles and responsibilities, mechanisms for timely and constructive feedback and ensuring resource availability (including adequate nursing and anesthesia support). Additionally, an effective pathway involves protocols for specialist integration into local teams and, as noted by study respondents, satisfactory remuneration and appropriate reimbursement for costs associated with outreach work.

Ideally, the mutual benefit for rural and regional sites comes from exposure to increased procedural volume and scope for healthcare providers in the rural hospital and the opportunity for specialists to increase their procedural volume. Rural physicians and nurses are better able to maintain existing skills and can develop new skills. Specialists gain exposure to different practice settings and patients and can increase their income. There is also a systems advantage in providing low acuity care with low acuity patients in smaller sites to stem patient flow into larger centres, thereby allowing the room for more complex care. A truly distributed model of surgical and procedural care can also contribute to meeting surgical benchmarks by filling unused rural OR time. An example of the success of this approach was observed in some RSON hospitals that saw a threefold increase in OR dental procedures between 2017 and 2022.

To our knowledge, this is the first study from Canada to investigate the experiences of multiple specialists who provide outreach services in rural communities. Even though the sample size is small, a moderate response rate and diversity of respondents in characteristics such as practice experience was achieved.

## Conclusion

The feedback provided by study respondents give us direction in how to strengthen network models of care. The following recommendations will contribute to fully actualizing the potential of the outreach specialist model.

1. Regional Health Authorities should commit to regionalized procedural care from clinical and administrative perspectives by ensuring incentives and supporting outreach care at both rural and regional sites. This involves establishing benchmarks for outreach care based on rural population needs.
2. As one enabler of effective outreach care is rural site stability, efforts should be made to ensure adequate human resource and infrastructure funding.
3. Outreach care must be comprehensively supported through funding mechanisms that are easy to access and which compensate not only for clinical care but also for the contextual reality of lost clinical time as well as travel and travel related costs. Although BC currently has a funding stream to support outreach care, the Northern and Isolation Travel Assistance Outreach Program should be reviewed to ensure fit-for-purpose for the specialists and administrative structures supporting them.
4. Rural sites should regularly engage visiting specialists in the context of quality improvement, formally and informally, to understand their perspectives and experiences with the goal of iterative improvement.

Overall, specialists in this study had a positive outreach experience, but indicated they can be better supported to provide high-quality care through investments in infrastructure and health human resources. These investments are likely to yield a positive return on rural health. More research, including from other jurisdictions in Canada, can help us to better understand the facilitators and barriers to specialist outreach work.

## Supporting information

S1 Fig Quality of care at RSON hospitals–Teamwork (n = 21). (TIF)

S2 Fig Integration into the local RSON hospital team over the past six months (n = 20). (TIF)

S1 Data Survey responses with identifying information removed. (XLSX)
