# Supplementary figures and images for: The motivations and experiences of specialists who provide outreach services in rural operating rooms: A survey study from British Columbia
Source: PLoS One. 2024 Mar 27;19(3):e0298757. doi: 10.1371/journal.pone.0298757 (PMC10971771; doi:10.1371/journal.pone.0298757)

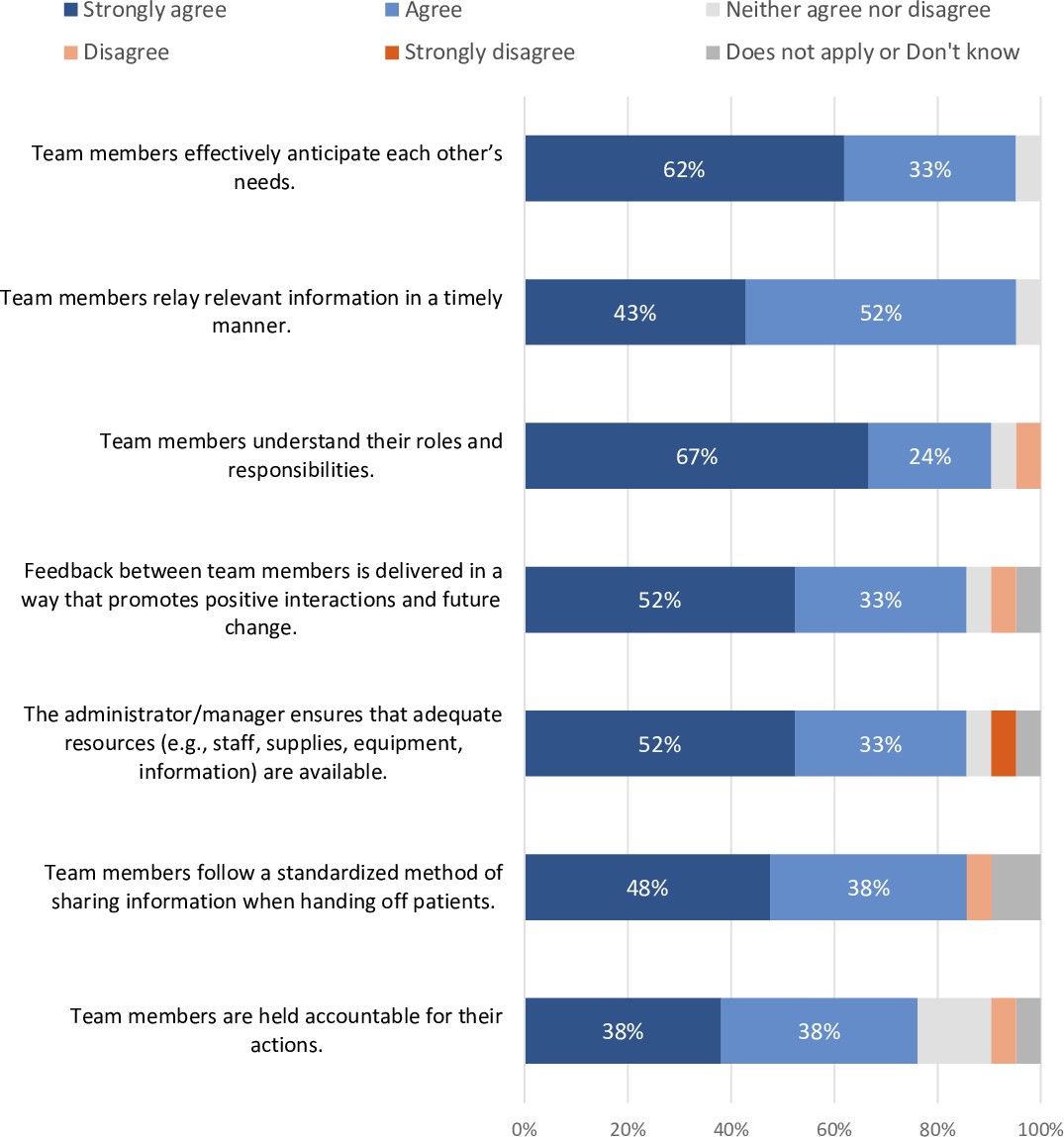

Supplement: S1 Fig — (TIF) [file pone.0298757.s001.tif]

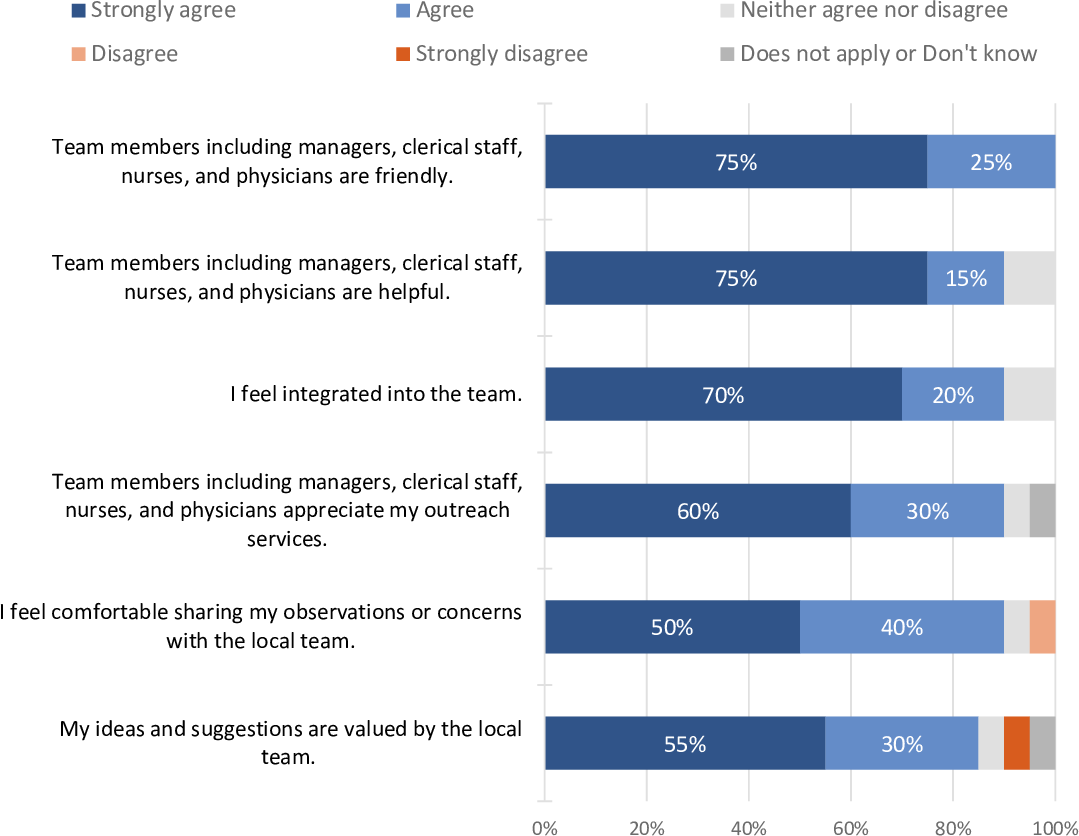

Supplement: S2 Fig — (TIF) [file pone.0298757.s002.tif]
